# Targeting cancer cachexia: Molecular mechanisms and clinical study

**DOI:** 10.1002/mco2.164

**Published:** 2022-09-10

**Authors:** Yong‐Fei Wang, Zi‐Yi An, Dong‐Hai Lin, Wei‐Lin Jin

**Affiliations:** ^1^ The First Clinical Medical College of Lanzhou University Lanzhou China; ^2^ Institute of Cancer Neuroscience Medical Frontier Innovation Research Center The First Hospital of Lanzhou University Lanzhou China; ^3^ Key Laboratory for Chemical Biology of Fujian Province MOE Key Laboratory of Spectrochemical Analysis and Instrumentation College of Chemistry and Chemical Engineering Xiamen University Xiamen China

**Keywords:** cancer cachexia, innervation, interorgan communication, metabolism, muscle wasting, exercise

## Abstract

Cancer cachexia is a complex systemic catabolism syndrome characterized by muscle wasting. It affects multiple distant organs and their crosstalk with cancer constitute cancer cachexia environment. During the occurrence and progression of cancer cachexia, interactions of aberrant organs with cancer cells or other organs in a cancer cachexia environment initiate a cascade of stress reactions and destroy multiple organs including the liver, heart, pancreas, intestine, brain, bone, and spleen in metabolism, neural, and immune homeostasis. The role of involved organs turned from inhibiting tumor growth into promoting cancer cachexia in cancer progression. In this review, we depicted the complicated relationship of cancer cachexia with the metabolism, neural, and immune homeostasis imbalance in multiple organs in a cancer cachexia environment and summarized the treatment progress in recent years. And we discussed the molecular mechanism and clinical study of cancer cachexia from the perspective of multiple organs metabolic, neurological, and immunological abnormalities. Updated understanding of cancer cachexia might facilitate the exploration of biomarkers and novel therapeutic targets of cancer cachexia.

## Graphical abstract

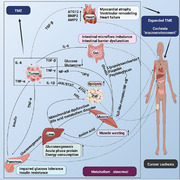

**Abbreviations**
4EBP1: 4E‐binding protein 1
ACTRIIB: activin receptor IIB
APR: acute phase protein
ATGL: adipose triglyceride
BAT: brown adipose tissue
CRP: C‐reactive protein
ECM: extracellular matrix
GDF15: growth differentiation factor 15
GFRAL: glial cell‐derived neurotrophic factor family receptor alpha‐like
gp130: glycoprotein 130
HPA: hypothalamus–pituitary–adrenal axis
HPG: hypothalamus–pituitary–gonad axis
HSL: hormone‐sensitive lipase
HSP70: heat shock proteins 70
HSP90: heat shock proteins 90
IGF‐1: insulin‐like growth factor‐1
IL18: interleukin‐18
IRS‐1: insulin receptor substrate 1
JAK: Janus kinase/signal transducer
LLC: Lewis lung cancer
MAPK: mitogen‐activated protein kinase
MDSCs: myelogenous suppressor cells
mTOR: Mechanistic target of rapamycin
NF‐κB: nuclear factor‐kappa B
NPY: neuropeptide Y
PDAC: pancreatic ductal adenocarcinoma
PI3K: phosphatidylinositol‐3‐kinase
POMC: proopiomelanocortin
PTHrP: parathyroid hormone‐related protein
SNS: sympathetic nervous system
STAT: activator of transcription
TGF‐β: transforming growth factor‐β
TGR5: Takeda G‐protein‐coupled receptor 5
TME: tumor microenvironment
TNF‐α: tumor necrosis factor‐α
UCP2: uncoupling protein2
WAT: white adipose tissue

## 1 INTRODUCTION

Malignant tumors impose great burden on human health. In the past years, tumor treatment strategies have shifted from tumor centric into tumor microenvironment (TME) centric. As the recognition of tumor and whole body as extended “seed and soil,” the involvement of cancer‐organ crosstalk and interorgan signal crosstalk in tumor growth and progression has been proposed.^1^ Interstitial cells in TME and the balance of the whole organism are reshaped in cancer, consequently promoting the accumulation and spread of cancer cells, reducing the ability of the immune system to combat tumor growth, and directly leading to cancer‐related lethality.

The term “cachexia” is derived from the Greek words kakos (bad) and hexis (habit).^2^ Cachexia is a term that has been used for a long time to describe a state of wasting due to malnutrition.^3^ Cachexia is a malnutrition associated with chronic diseases^4^ and a serious but underrecognized consequence of many chronic diseases.^5^ Such as cancer, chronic heart failure, chronic obstructive pulmonary disease, chronic kidney disease, rheumatoid arthritis, and human immunodeficiency virus infection.^5^, ^6^, ^7^ The prevalence of cachexia in these diseases ranges from 5 to 90%.^5^, ^7^, ^8^, ^9^, ^10^, ^11^, ^12^ However, cachexia is associated particularly with cancer.^5^ Therefore, cancer cachexia has received increasing attention in recent years.

Cancer cachexia is the result of most malignant tumors and imposes a major burden on the global health care system, exerting far‐reaching negative impact on the treatment response, quality of life, and long‐term survival of patients.^13^, ^14^, ^15^, ^16^, ^17^, ^18^, ^19^ The incidence of cachexia in common malignant tumors ranges from 20 to 90% (Table 1). The signal crosstalk between tumors and multiple organs induce the homeostatic imbalance in multiple organs,^20^ and multiple organs will lose control of tumor growth. For example, tumor growth leads to the dysfunction of organ metabolism and neural and immune processes and leads to the destruction of homeostasis and the loss of inhibitory effect on tumor growth.

**TABLE 1:** Incidence of cachexia in different tumors

Tumor	Incidence of cachexia (%)	(Reference)
Gastric cancer	85	^21^
Pancreatic cancer	83	^21^
Nonsmall cell lung cancer	61	^21^
Small cell lung cancer	57	^21^
Advanced head and neck cancer	57	^22^
Prostate cancer	56	^21^
Colon cancer	54	^21^
Unfavorable non‐Hodgkin's lymphoma	48	^21^
Sarcoma	40	^21^
Acute non‐lymphocytic leukemia	39	^21^
Breast cancer	36	^21^
Favorable non‐Hodgkin's lymphoma	31	^21^
Hepatocellular carcinoma	25	^23^

However, given that cachexia is often caused by advanced tumors,^24^, ^25^ its treatment might be ignored. In addition, the mechanism of cachexia is unclear.^26^, ^27^, ^28^ No exact diagnostic criteria and effective treatment strategies for cancer cachexia have been established.^29^ An in‐depth understanding of the relationship between tumors and multiple organs is of great significance to understanding of the progression of cancer cachexia and development of interventions or treatment strategies. Herein, we discuss the role of multiple organs crosstalk in the occurrence and development of cancer cachexia and explore potential diagnostic markers and treatment targets. Cancer cachexia is now considered to be a condition caused by metabolic, immunological, and neurological abnormalities rather than mere nutritional abnormalities.^4^, ^30^, ^31^ We tried to explore the molecular mechanism and clinical research of cancer cachexia from the perspective of multiple organs metabolic, neurological, and immunological abnormalities. Despite that no treatment method has been approved,^32^ the symptomatic treatment of cancer cachexia can improve the quality of life and survival rate of patients. Furthermore, we list current treatment strategies of cancer cachexia.

## 2 CLINICAL CHARACTERISTICS OF CANCER CACHEXIA

Cachexia is a syndrome that causes severe debilitation and usually associated with cancer; it is characterized by muscle loss with or without corresponding adipose tissue loss.^33^ The main clinical manifestations of cancer cachexia include anorexia, weight loss, and multiple organs dysfunction. Its complex clinical symptoms affect more than 50% of patients with cancer and 60–80% of patients with advanced cancer and causes the death of at least 20% of patients with cancer.^34^ The incidence of cachexia varies with tumor type^21^, ^22^, ^23^ (Table 1), and the factors leading to changes are unclear, which may be closely related to tumor stage, sex and age of patients, genetic risk factors, complications, and treatment response. The prevalence of cancer cachexia may be related to patient genotype, and some candidate genes have been used in exploring and elucidating individual differences in cachexia susceptibility.^35^

**FIGURE 1:**
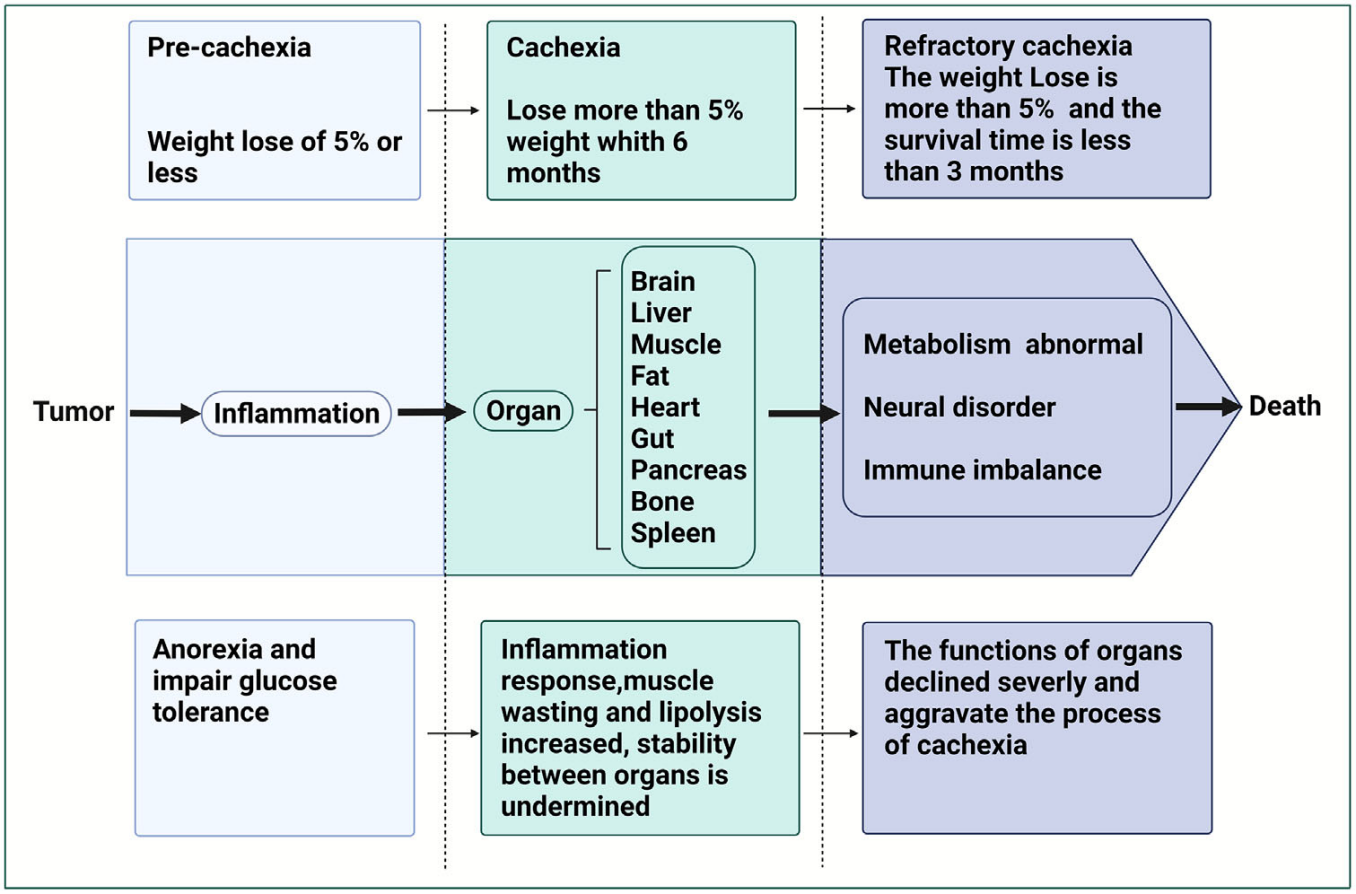
Organ changes in the journey of cancer cachexia. Cancer cachexia can be classified into three stages: precachexia, cachexia, and refractory cachexia. In the progression of cancer cachexia, the functions and states of organs gradually change in metabolism, neural, and immune processes. There is a complex and changeable relationship between organs and cancer cachexia. Although muscle wasting and lipolysis is the main feature of cancer cachexia, tumor‐ and host‐derived factors and systemic inflammation affect many other organs, such as the liver, heart, intestine, pancreas, brain, bone, and spleen. The functions of multi‐organ decline, and the process of cachexia is affected. Adapted from Ref. 36. Created with BioRender.com.

Based on the high incidence and mortality of cachexia in patients with cancer, studying the clinical stages of cancer cachexia and changes in organs in each period is important, and treatment should be established according to different stages and organ states. According to severity, cancer cachexia can be classified into three stages: precachexia, cachexia, and refractory cachexia^36^ (Figure 1), which occur in succession. However, not every patient with cancer cachexia goes through three stages.^36^ Weight loss of 5% or less, accompanied by anorexia and impaired glucose tolerance, is known as the precachexia, which tends to involve only metabolic changes.^4^, ^37^ Patients with cachexia are characterized by involuntary weight loss of more than 5% within 6 months and cannot be completely reversed by traditional nutritional support. In refractory cachexia, patients have limited benefits from related treatments due to active catabolic levels and usually have a survival period of less than 3 months. At this stage, the only goal of treatment is to alleviate a patient's suffering.^38^ The stage of cancer cachexia is directly related to weight loss and survival rates of patients. In the three stages of cancer cachexia, the development of multiple organs and cancer cachexia is a relationship of growth and decline. In precachexia, organs and tumors are in a relatively balanced stage and anorexia and impair glucose tolerance are the early symptoms in precachexia.^25^, ^37^ Through mutual communication and cooperation, multiple organs can resist the abnormal changes caused by cancer cachexia. Hence, a patient's weight does not exhibit large loss, and patients can maintain general physical activities. During cachexia, the functions of organs declined. Increased release of inflammatory factors enables cancer cachexia to utilize signal communications between organs. Decline in the function of one organ can lead to the abnormality of another organ through signal communication, stability in organs is undermined, and muscle wasting and lipolysis increase. Inflammatory factors released by tumors and hosts are the main reasons for the progression of the disease. In refractory cachexia, catabolism is exuberant, signal communication between organs is interrupted or becomes a tool to promote the development of cachexia. The function of each organ drops in a straight line, and no synergy to fight cancer is present.^25^, ^39^ Organ failure releases related factors, which in turn aggravate the progression of cancer cachexia (Figure 1). Communications between tumor cells, TME, and multiple organs promote the occurrence of cachexia. Next, we will describe the typical signal communications between multiple organs during cancer cachexia and how they affect the course of cancer cachexia. We also talked about the interaction between TME and cancer cachexia. The communications among tumor cells, TME and multiple organs together constitute the macroenvironment of cancer cachexia.

## 3 MULTIPLE ORGANS METABOLIC DISTURBANCE IN CANCER CACHEXIA

Tumor have metabolic crossover with cells in their surrounding environment and affect the metabolism of the whole body. Metabolic changes associated with tumorigenesis allow transformed cells to settle in different tissues by evading homeostatic defense and using internal signal mechanisms. When cancer cells leave original tissue and settle in other organs, they may be potentially restricted by the tissue microenvironment of a metastatic organ.^40^ However, metastatic cancer cells still maintain the metabolic characteristics of origin tissues and reuse these characteristics to resist the restriction of metastatic organs. Finally, metastatic cancer cells adapt to the metabolism of metastatic organs, thus promoting tumor growth. The most prominent manifestation of tumor systemic metabolism is cachexia in multiple organs catabolism. In the early stage of a tumor, multiple organs begin to inhibit tumor progression. When the tumor develops and enters the cachexia stage, multiple organs show metabolic disorders.

### 3.1 Crosstalk between liver and muscle wasting

The liver plays a vital role in glucose, adipose, and protein metabolism and is essential to the body's ability to cope with changes in nutritional status. In view of the key functions of the liver, decline in liver metabolism is first sign of metabolic diseases,^41^ and the liver is one of the first organs to change in the form of metabolic reprogramming when cancer cachexia occurs. In the early stage of cancer cachexia, the liver consumes energy through hepatomegaly, production of acute phase proteins (APRs), and increased gluconeogenesis. Increase in visceral organ and tumor mass indicates an increase in resting energy consumption, which may be related to weight loss associated with cachexia. In cancer cachexia, many inflammatory factors are produced in the liver and aggravate the symptoms of cancer cachexia. For example, interleukin‐6 (IL‐6) secreted by hepatocytes can promote muscle wasting, the canonical IL‐6/JAK (Janus kinase)/STAT(signal transducers and activators of transcription) and fibroblast growth factor/p38 mitogen‐activated protein kinase (MAPK) signaling pathways can cause muscle wasting.^42^ IL‐6 secreted by hepatocytes can also lead to increased lipolysis. In a colorectal mouse model, it was shown that IL‐6 activates thermogenesis and increases fat consumption by increasing the expression of Uncoupling Protein 1 and genes associated with fatty acid oxidation.^43^ Some anti‐inflammatory factors such as IL‐4 are downregulated in the liver.^44^ These inflammatory reactions reduce the survival rate of patients with cancer cachexia, and thus their specific mechanism need to be further studied.^45^

As a tumor progresses, it increases its glucose and glutamine consumption for its growth, depriving muscles of nutrition, causing muscles to decompose into amino acids, and promoting gluconeogenesis in the liver and APR synthesis in hepatocytes. APR include C‐reactive protein (CRP), serum amyloid A, α1‐antitrypsin, fibrinogen, α1‐acid glycoprotein, haptoglobin, α2‐macroglobulin, ceruloplasmin, and complement factors B and C3,^46^ but the serum protein in plasma will decrease significantly and the ratio of albumin: CRP has been used successfully as a prognostic indicator in the Glasgow Prognostic Score.^47^ So is there such a possibility, this increase in APRs accelerates the decomposition of muscle proteins for synthetic protein use in the liver, which promotes the transfer of nitrogen from muscle to liver in the form of amino acids, further aggravating muscle wasting,^48^, ^49^, ^50^, ^51^ but there is no accurate relevant experimental evidence.^20^, ^52^ In fact, different protein metabolic pathways was observed in liver and skeletal muscle in tumors. Liver protein synthesis is a very active process and protein degradation plays a major role in skeletal muscle in tumors. Therefore, these protein metabolic differences promote the transport action of amino acids from skeletal muscle to liver in skeletal muscle and liver.^20^, ^53^ In general, circulating factors released by tumors and host cells indirectly affect muscle health, increasing energy consumption and producing large amounts APR that promote inflammation.

The crosstalk between liver and muscle during cancer cachexia is manifested in bile acid metabolism. Bile acids are well‐known regulators of inflammation and energy homeostasis,^54^, ^55^, ^56^ two key features of cancer cachexia. Cholestasis occurs in cancer cachexia, promotes the development of liver inflammation, and promotes the decomposition of muscles and adipose.^57^ Muscle wasting caused by increased bile acid in cancer cachexia is mainly related to the activation of Takeda G‐protein‐coupled receptor 5 (TGR5) in muscle cells. An animal study showed that bile acids promote the activation of intracellular thyroid hormones through TGR5, and thereby increase the energy consumption of human adipocytes and skeletal muscle cells.^58^ Thus targeted drugs for TGR5 may play a role in the treatment of cancer cachexia.^59^ There are many therapeutic targets for bile acid in cancer cachexia, one study show the use of drugs such as tauroursodeoxycholic acid, to target bile acid metabolism may be helpful to the treatment of cancer cachexia.^60^

The energy of muscle and adipose tissue decomposition during cancer cachexia is not fully utilized by the body. This phenomenon is related to energy waste caused by the accumulation of cardiolipin in liver mitochondria during cancer cachexia and is characterized by decrease in oxidative phosphorylation efficiency.^61^, ^62^, ^63^, ^64^ This energy waste is related to the increased levels of tumor necrosis factor‐α (TNF‐α) and high energy consumption conditions.^64^ The insufficient utilization of energy in turn stimulate the body to constantly extract energy from muscles and adipose tissues, creating a vicious cycle of energy waste.

Tumors influence the metabolic state of an organism by scrambling normal circadian oscillations in metabolic gene expression in the liver.^65^ In the early stage of cancer cachexia, the liver compensates the energy consumption of the body by increasing of its volume, delaying the progress of cachexia. However, with the aggravation of cachexia, beyond the compensatory capacity of the liver, abnormal liver metabolism directly or indirectly leads to muscle wasting, which in turn accelerates the abnormal metabolism of the liver.

The complex metabolic relationship between the liver and muscles during cancer cachexia has an important influence on the occurrence and development of the disease, but the specific mechanism remains to be further studied.

### 3.2 Crosstalk between muscle wasting and lipolysis

Muscle wasting is the most important feature of cancer cachexia and reduces the mobility of patients.^66^ It can reduce the tolerance of tumor patients to radiotherapy and chemotherapy.^67^, ^68^, ^69^, ^70^ Transforming growth factor‐beta (TGF‐β), myostatin, activin, insulin‐like growth factor‐1 (IGF‐1)/phosphatidylinositol‐3‐kinase (PI3K)/AKT, and JAK‐STAT signaling pathways are known to underlie muscle wasting.^71^ The catabolism of skeletal muscle proteins in cancer cachexia is caused by three major proteolytic pathways, including the ubiquitin–proteasome system, the calcium‐activated system, and the autophagy–lysosome pathway.^72^, ^73^ The inhibited pathways of protein synthesis are energy‐dependent AMP–activated protein kinase–mechanistic target of rapamycin (mTOR) pathway and insulin‐dependent IGF‐1–AKT–mTOR pathway.^73^ It has been documented that various cytokines and cachexia factors can regulate the loss of skeletal muscle mass. These proinflammatory and cachexia factors produced by tumor cells are considered to play an important role in the occurrence and development of cachexia. For example, proinflammatory cytokines, such as TNF‐α, IL‐1, and IL‐6, promote the activation of transcription factors associated with muscle wasting and play a key role in the pathological mechanism of cachexia.^30^, ^74^ These factors act individually or interaction (IL‐6 and TNF‐α) as drivers of systemic inflammation. Proinflammatory factors of cancer cachexia genesis are involved in the activation of various downstream transcription factors and molecules in muscle. TNF‐α induces catabolism of myofibrillar proteins in myotubes by activating nuclear factor‐kappaB (NF‐κB). Activated NF‐κB increases the transcription of genes such as myoring finger protein‐1, which encodes an enzyme in the ubiquitin proteasome pathway‐E3 ligase. E3 ligase specifically degrades myofibrillar proteins such as actin, myosin heavy and light chains, as well as troponin‐I, and promotes lean body weight loss during cancer cachexia.^75^ Similarly, IL‐6 induces apoptosis of skeletal muscle cells by activating JAK/STAT and MAPK to increase caspase activity. In addition to TNF‐α and IL‐6, recent preclinical studies in mice of anorexia–cachexia syndrome have shown that higher levels of IL‐10 inhibit skeletal muscle protein synthesis by increasing Myc levels and activating mTOR signals. The inhibition of these factors can restore muscle mass and prevent tumor‐related cachexia.^76^ However, the molecular regulation mechanism of cancer cachexia‐induced muscular wasting mainly comes from preclinical models, and how these factors can be transformed into a clinical environment needs to be further explored.

Lipolysis is increased in cancer cachexia. Although increased lipolysis is not necessary in cancer cachexia, the disorder of lipid metabolism and increase in lipolysis factors in the circulation occur in the early stage of cancer and increase with the deterioration of tumors.^77^, ^78^, ^79^ This finding suggests that lipolysis factors in the early stage of circulation can be used as early diagnostic markers of cancer cachexia. Adipose tissues can secrete factors that promote systemic inflammation, such as TNF‐α, IL‐6, and IL‐18,^80^ which in turn lead to lipolysis. TNF‐α promotes lipolysis by activating MAPK (p44/42) and Jun N‐terminal kinase pathway. A recent study found that leukemia inhibitory factor and ciliary neurotrophic factor from the IL‐6 family activate JAK/STAT signaling pathway and induce lipolysis by binding receptor leukemia inhibitory factor α and coreceptor glycoprotein 130 (gp130). The activation of JAK/STAT leads to the upregulation of adipose triglyceride (ATGL) expression.^51^, ^81^ In addition to inflammatory factors, in the context of cancer cachexia, catecholamines and corticosteroid hormones released by tumor and host cells lead to a hypermetabolic state and play an important role in fat consumption.^82^ It has been shown that the tumor‐derived factor zinc‐a2‐glycoprotein, known as a lipid mobilizing factor, increases fat consumption in mice with cachexia.^83^ Spiegelman et al. identified that parathyroid hormone‐related peptide (PTHrP) plays an important part in the browning of adipose tissue. Treating mice with a thyroid hormone receptor agonist leads to activation of thermogenesis in subcutaneous white adipose tissue (WAT), which shows that hyperthyroidism can lead to adipose tissue browning.^82^, ^84^ In addition to lipolysis, loss of adipose tissues may be related to the decreased ability of adipocytes to synthesize and store lipids. The decreased expression of adipogenic transcription factors has been observed in many cancer cachexia models and may be related to the decrease in the volumes of adipocytes and high expression of TNF‐α.^85^ It is worth mentioning that some adipokines have inhibitory effects on the development of cancer cachexia. It is worth mentioning that some adipokines have inhibitory effects on the development of cancer cachexia. For example, ghrelin secreted by the gastrointestinal tract is a newly discovered endogenous ligand of growth hormone secretagogue receptor.^86^ This ligand increases food intake by activating appetite‐stimulating mediators, such as neuropeptide Y (NPY), in the hypothalamus.^87^ Except its appetite‐promoting effect, ghrelin inhibits protein degradation promoted by cytokines, thereby inhibiting muscle wasting during cancer cachexia.^87^, ^88^, ^89^ In addition, ghrelin can inhibit skeletal muscle cell apoptosis induced by adriamycin (an antineoplastic drug).^90^

In the context of cancer cachexia, there is a close relationship between muscle wasting and lipolysis, and adipose tissue can indirectly affect skeletal muscle through the tumor–adipose tissue–muscle axis. For example, in the Lewis lung cancer (LLC) model, tumor‐derived parathyroid hormone‐related protein (PTHrP) binds to the parathyroid hormone/PTHrP receptor on white adipocytes to promote WAT browning, and neutralization of PTHrP indirectly protects skeletal muscle mass and function.^84^ In patients with cancer cachexia, lipolysis occurs earlier than muscle consumption,^91^ and muscles can increase the rate of fatty acid metabolism and level of p38 oxidative stress in skeletal muscle by secreting inflammatory factors before muscle wasting.^92^, ^93^ Inhibiting lipolysis seems to have a protective effect on muscle wasting. For example, inhibiting ATGL and hormone‐sensitive lipase (HSL) can also reduce muscle wasting,^94^ which reflects the close relationship between muscle and adipose in cancer cachexia. In addition to the protective effect of adipose tissue on muscle, TNF‐α secreted by adipose tissues can significantly induce the expression of uncoupling protein2 and uncoupling protein3 in skeletal muscles, which promote thermogenesis and accelerate skeletal muscle decomposition. Myostatin is a cachexia factor secreted by skeletal muscle, adipose tissue, and tumor cells.^95^ Myostatin acts through activin receptor type II‐mediated signaling and regulates muscle wasting.^96^ Muscle wasting can promote lipolysis, and the muscle‐derived factor irisin can induce the browning of WAT and increases thermogenesis.^97^

Given that adipose and muscle mass can be used in predicting the survival rates of patients with cancer cachexia,^98^, ^99^ studying the relationship between muscle wasting and lipolysis during cancer cachexia and exploring methods to inhibit muscle wasting and lipolysis are meaningful. Related treatments are under study currently.^13^, ^25^, ^31^, ^72^, ^74^, ^100^, ^101^, ^102^, ^103^, ^104^, ^105^, ^106^, ^107^, ^108^, ^109^, ^110^, ^111^, ^112^, ^113^, ^114^, ^115^, ^116^, ^117^, ^118^

### 3.3 Cardiac metabolism and cancer cachexia

In addition to the loss of skeletal muscles and adipose tissues, cancer cachexia is associated with myocardial atrophy, which is accompanied by cardiac remodeling and dysfunction.^119^, ^120^ In a variety of cancer cachexia models, the heart atrophies obviously. Echocardiographic evidence shows that impaired cardiac function and systolic and diastolic dysfunction was occurred, followed by increase in myocardial fibrosis^120^ and heart failure occurred. Heart failure in the context of tumors has attracted considerable interest because cachexia can easily lead to heart failure, which in turn aggravates cachexia and muscle wasting.^121^, ^122^, ^123^ Heart failure accounts for a large proportion of deaths caused by cancer cachexia.^119^, ^124^ The mechanism of heart failure in cancer cachexia is unclear. The mRNA levels of autophagy‐related protein 12, adenovirus E1B 19‐kDa‐interacting protein 3, and ampelopsin 2 increase in the myocardium of a mouse cachexia model. The heart shows atrophy and dysfunction, accompanied by cardiac necrosis, inflammation, and fibrosis.

Different from the previous thought that myocardial remodeling in cancer cachexia is caused by toxicity of anticancer therapy,^125^ mounting evidence supports the causal relationship between cancer cachexia and cardiac remodeling.^126^ Cardiac remodeling may be related to TGF‐β released during cancer cachexia. The tumor–host interaction causes insulin resistance and inhibit the myocardial protein synthesis pathway IGF‐1–PI3K–Akt–MTOR. Tumor proliferation requires increased adenosine triphosphate, activates AMPK, and inhibits mTOR. mTOR reduces the expression of S6K1 and 4E‐binding protein 1(4EBP1), leading to reduced synthesis of myocardial proteins. In addition, myostatin activates the activin receptor type IIB (ACTRIIB) receptor, causing smad2/3 activation to inhibit AKT and reduce cardiac protein production.^127^ Similar to skeletal muscles,^124^ the inhibition of NF‐κB improves cardiac atrophy and function in mice with CT26 cancer cachexia. As mentioned above, TNF‐α induces catabolism of myofibrillar proteins in myotubes by activating NF‐κB. Activated NF‐κB increases the transcription of genes such as myoring finger protein‐1, which encodes an enzyme in the ubiquitin proteasome pathway–E3 ligase and eventually leads to myocardial atrophy. Therefore, it is plausible that NF‐κB inhibition prevented the upregulation of MuRF1 and Atrogin‐1. Recent studies have discovered a new drug that can inhibit NF‐κB by targeting the IκB kinase, thereby improving myocardial atrophy caused by tumor cachexia,^128^ but some differences in mechanism between cardiac and skeletal muscle wasting caused by cancer cachexia have been found. Mühlfeld et al.^129^ observed that cardiac remodeling induced by cachexia is related to the impairment of cardiac nerve function in LLC model suggested that the reason is the decreased expression of nerve growth factor in tumor‐bearing mice, which is not observed in skeletal muscles. A signal crosstalk between heart and cancer cells occurs during cancer cachexia, and metabolic reprogramming occurs in the heart under the action of related factors secreted by a tumor or host.^130^ Remodeled heart and cancer cells exhibit intimate crosstalk via multiple secreted factors,^131^ which aggravate the cachexia process. The specific mechanism of cardiac atrophy in cancer cachexia is far from clear, and future studies on changes in the cardio‐cerebral and cardiac–gut axes in cancer cachexia may discover a new mechanism for cardiac atrophy.

### 3.4 Pancreatic metabolism and cancer cachexia

Pancreatic metabolism dysfunction plays an important role in the occurrence and development of cancer cachexia. Cancer patients show impaired glucose tolerance and insulin resistance,^132^ and insulin resistance increases gradually with the development of cachexia.^72^, ^133^ Insulin resistance has been shown to cause cachexia in mouse and Drosophila tumor models. Studies using Drosophila and Drosophila melanogaster models have shown that ImpL2 secreted by tumor, a homologue of IGF binding protein and a potent antagonist of insulin signaling, leads to systemic metabolic impairment and muscle wasting.^134^ Impaired pancreatic insulin secretion also leads to insulin resistance and triggers muscle wasting in the Walker 256 cancer cachexia model.^135^ One of the factors inducing insulin resistance is TNF‐α, which directly disrupts insulin the signaling and activation of insulin receptor‐1.^136^ In cancer cachexia, insulin plays an important role in communications between the pancreas and liver and between the pancreas and tumors. Insulin resistance can promote gluconeogenesis in the liver and lead to muscle wasting by inhibiting protein synthesis. The amino acids released by muscle decomposition enter the blood circulation and promote the aerobic glycolysis of the tumor, which accelerate the tumor growth and the waste of energy. Insulin maintains the vitality of organs by taking up glucose in muscle, fat and other tissues. Insulin resistance inhibits the PI3K/AKT/mTOR pathway in cancer cachexia. Insulin or IGF‐1 binds to its receptor and induces glucose uptake in muscle tissue by activating the AKT/mTOR signaling pathway. In insulin resistance, insulin receptor tyrosine kinase activity is reduced, and insulin and IGF‐1 binding to the receptor is reduced, resulting in insufficient signal transduction pathways. Insulin pathway inactivation leads to inactivation of insulin receptor substrate 1 (IRS‐1).^137^ IRS‐1 inactivation leads to PI3K inactivation, reducing AKT phosphorylation. Reduced AKT phosphorylation activates the Forkhead box O3 protein, which translocates to the nucleus and activates protein degradation in muscle. In addition, reduced AKT phosphorylation leads to inactivation of mTOR, P70S6 kinase 1, and 4EBP1, preventing protein synthesis in cancer cachexia.^138^ AKT protein expression was inhibited in male CD2F1 mice with colon‐26 adenocarcinoma tumors. Inhibition of AKT protein leads to inactivation of AKT/mTOR signaling, which in turn leads to insulin resistance and muscle atrophy in mice. Like muscle tissue, insulin resistance can also increase fat consumption by inhibiting the PI3K/AKT/mTOR pathway in adipose tissue.^139^ Increased fat consumption is also associated with reduced glucose uptake by adipose tissue in the presence of insulin resistance.^140^ Studies have confirmed that in InsrP1195L/+ mice, insulin resistance decreased expression of phosphorylated AKT in WAT and liver tissues of InsrP1195L/+ mice.^141^ Furthermore, AKT2‐defcient null mice showed glucose intolerance and hyperinsulinemia. Insulin resistance and altered glucose homeostasis in cachexia play a key role in increasing lipolysis. Overall, insulin resistance increases the level of insulin in circulation, and insulin is a growth factor that can promote tumor growth, which accelerates the process of cachexia. Therefore, maintaining glucose homeostasis and improving insulin sensitivity are the keys to suppressing muscle wasting in cancer cachexia.

Based on some of the same metabolic characteristics between diabetes and cancer cachexia, metformin reduces cancer‐induced muscle wasting in mice.^142^ Rosiglitazone can prevent weight loss and improve the survival rate of cancer cachexia in rat models.^143^ In a randomized controlled study of patients with cachexia, compared with treatment without insulin, treatment administering a small amount of insulin daily increases metabolic efficiency during exercise by increasing the whole body fat content and significantly improves survival rate.^144^ These studies reflect the complex relationship between cancer cachexia and the pancreas, especially the effect of insulin secreted by the pancreas on the metabolism of multiple organs in cancer cachexia.

### 3.5 Intestinal metabolism and cancer cachexia

The number of microorganisms living in the intestinal tract is more than 10,^14^ the number of genomes is 100 times that in the human genome,^145^ and the number of bacteria is equal to that of human cells.^146^ Thus, the intestinal microflora plays an essential role in the occurrence and development of cancer cachexia.^147^ A recent study showed that increased abundance of enterobacteriaceae is positively associated with weight loss in the intestines of patients with cancer cachexia and decrease in the amount of short‐chain fatty acids is associated with the maintenance of the integrity of the intestinal immune system and intestinal barrier.^147^ In addition to intestinal microflora disorders, dysfunction of intestinal barrier driven by IL‐6 in cancer cachexia can exacerbate systemic inflammation and endotoxemia.^148^, ^149^, ^150^

The existence of the intestinal microflora–skeletal muscle axis has been reported,^151^ the metabolites produced by intestinal microflora can reach skeletal muscles and affect the energy consumption of muscle cells.^58^ The intestinal microflora can affect the bioavailability of amino acids, participate in the release of various metabolites (such as bile acid), and regulate the production of proinflammatory factors,^152^ which can affect muscle metabolism. Muscle wasting caused by the Toll‐like receptor (TLR)/NF‐κB pathway is related to the intestinal microflora skeletal muscle axis. Lipopolysaccharide, flagellin, and peptidoglycan released by intestinal bacteria can stimulate TLRs associated with cachexia in skeletal muscles, and TLRs can activate downstream NF‐κB factors, promoting muscle wasting.^151^

The brain–gut axis plays an important role in cancer cachexia. Intestinal autonomic regulation is the result of the joint actions of parasympathetic, sympathetic, and intestinal nerves.^153^ Communication between the brain and intestinal tract is bidirectional, the intestine synthesizes and secretes a variety of neuroactive substances that can cross the blood brain barrier to affect the brain, and neuroactive molecules originating from the brain can act on the intestine through the sympathetic and parasympathetic nervous systems or humoral pathways.^154^ These neuroactive substances include substance P, calcitonin gene‐related peptide, and NPY.^155^

**FIGURE 2:**
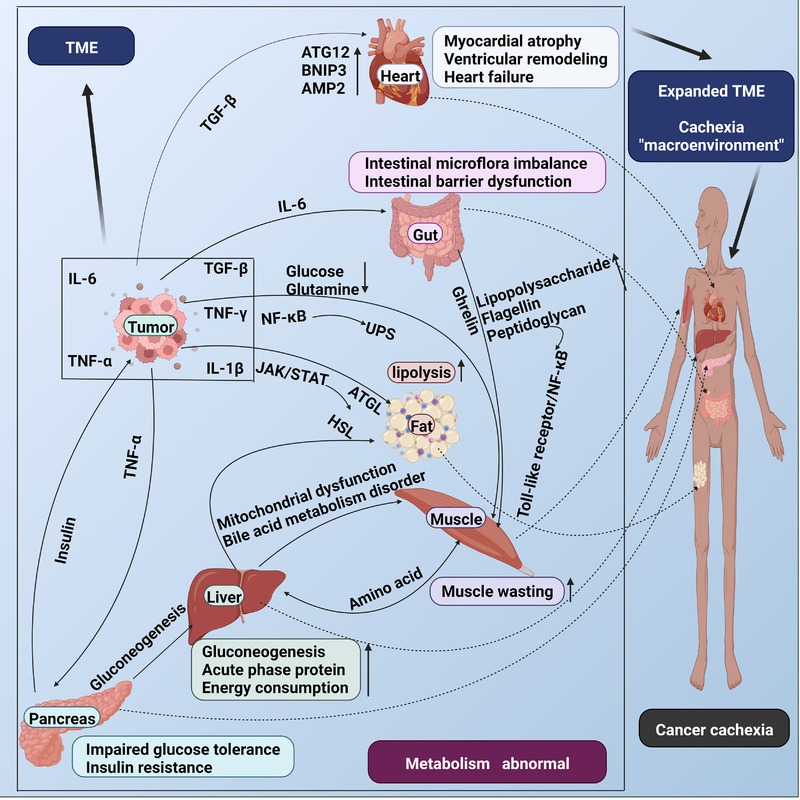
Crosstalk in the metabolism organs of cancer cachexia. Tumors and hosts secrete cytokines and inflammatory factors. Organs, such as the intestinal tract, can secrete ghrelin to promote appetite and reduce inflammatory reactions and muscle wasting, inhibiting progression of cancer cachexia. However, this negative feedback mechanism is active only in the early stage of tumors, and the amounts of inflammatory factors secreted eventually exceed the compensatory capacities of organs. Signal communication exists among metabolic organs, produces positive feedback, and accelerates the process of cachexia. Signal communications among tumor and muscle, fat, liver, heart, pancreas, and the intestinal tract aggravate the process of cachexia. Moreover, the functions of multiple organs gradually decline in the presence of metabolic disorders, which constitutes the “macroenvironment of cancer cachexia” along with the TME. The “macroenvironment” plays an important role in the occurrence and development of cancer cachexia. TME: tumor microenvironment; IL‐6: interleukin‐6; TNF‐α: tumor necrosis factor‐α; TGF‐β: transforming growth factor‐β; TNF‐γ: tumor necrosis factor‐γ; IL‐1β: interleukin‐1β; NF‐κB: nuclear factor nuclear factor‐kappa B; UPS: ubiquitin‐proteasome system; JAK: Janus kinase; STAT: signal transducer and activator of transcription; ATGL: adipose triglyceride lipase; HSL: hormone‐sensitive lipase; ATG12: autophagy related 12; BNIP3: adenovirus E1B 19‐kDa‐interacting protein 3; AMP2: ampelopsin 2. Created with BioRender.com.

In cancer progression, tumors and host cells secrete a variety of inflammatory factors, which cause intestinal metabolic disorders, but the gastrointestinal tract maintains homeostasis of the body and reduces the symptoms of cachexia by secreting ghrelin, promoting appetite and inhibiting cytokines by acting on the hypothalamus. These processes are manifestations of the competition between organs and tumors during cancer cachexia and indicate the role of signal communication between organs in the treatment of cancer cachexia. During cancer cachexia, the crosstalk of metabolic signals between organs and the TME constitutes the “macroenvironment of cancer cachexia,” which plays an important role in the occurrence and development of cancer cachexia (Figure 2).

## 4 MULTIPLE ORGANS NEURAL DISORDER IN CANCER CACHEXIA

**FIGURE 3:**
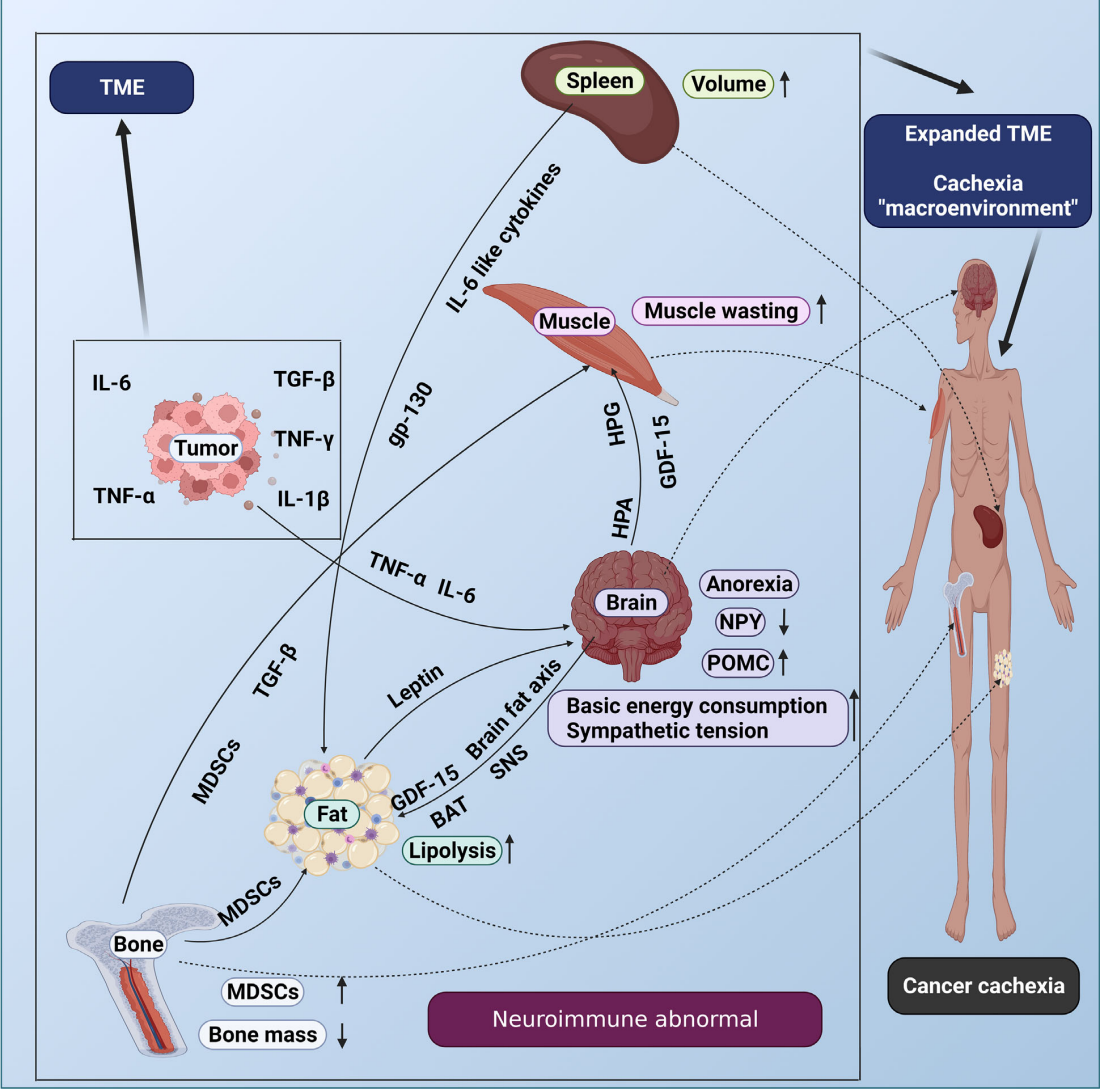
Crosstalk between neural and immune organs in cancer cachexia. Neuroregulation and immunomodulation play an important role in the process of cancer cachexia. Signal communications among tumors, muscles, fat, brain, bone, and spleen aggravate the process of cachexia. The crosstalk of the nerve and immune signals of organs and TME constitute the “macroenvironment of cancer cachexia,” which promotes cancer cachexia progression. TME: tumor microenvironment; IL‐6 interleukin‐6: TNF‐α tumor necrosis factor‐α; TGF‐β: transforming growth factor‐β; TNF‐γ: tumor necrosis factor‐γ; IL‐1β: interleukin‐1β; MDSCs: myeloid‐derived suppressor cells; gp‐130: glycoprotein‐130; GDF15: Growth differentiation factor 15; NPY: neuropeptide Y; POMC: proopiomelanocortin; HPA: hypothalamic–pituitary–adrenal; HPG: hypothalamic–pituitary–gonadal; SNS: sympathetic nervous system; BAT: brown adipose tissue. Created with BioRender.com.

The occurrence and development of tumors is closely related to the nervous system, which can affect tumors and innervate various targets in tumor tissues directly through sympathetic, parasympathetic, and sensory nerves. Moreover, it can regulate the activities of endocrine glands (such as adrenal glands), immune organs, and microbial flora indirectly. Tumors can directly affect brain activity through soluble mediators released by cells in the TME and can indirectly affect brain activity by altering metabolism. The metabolic effects of tumors are related to changes in hypothalamic function (such as hypothalamic inflammation) and may lead to the energy balance and development of cancer cachexia. The inhibition of the adverse effects of tumors on the brain may be helpful to the treatment of cancer cachexia.^156^ Metabolic changes caused by tumors stimulate the hypothalamus through inflammatory mechanisms, which respond through the autonomic nervous system and normalize peripheral physiology through norepinephrine or acetylcholine signals, but the hypothalamus cannot effectively perform this function due to the continuous stimulation of tumors.^157^ Signal communications between the hypothalamus and other organs play an important role in the occurrence and development of cancer cachexia (Figure 3).

### 4.1 Central nervous system and cancer cachexia

Increasing evidence shows that the central nervous system plays a decisive role in controlling the pathogenesis of cachexia by recognizing cytokines as molecular signals of disease;^158^, ^159^ the central nervous system can receive and amplify the roles of cytokines.^160^, ^161^ The hypothalamus is an important component of the structure of brain, which can accept a variety of internal and external stimuli and regulate the balance response in the body.^160^, ^162^, ^163^ In the context of cancer, hypothalamic neurons can sense many changes in physiological signals, including inflammatory cytokines (e.g., IL‐6)^164^ and endocrine hormones (e.g., leptin and ghrelin).^165^ These molecules play an important role by binding to receptors on hypothalamic neuron groups, such as proopiomelanocortin (POMC).^166^

The occurrence of anorexia in cancer cachexia is the result of multiple organs and multi‐system disorders. The mechanism of anorexia is affected by multiple factors, and cytokines secreted by tumors or hosts play a major role.^167^ The hypothalamus regulates food intake and body energy consumption by coordinating NPY and POMC,^168^ and leptin released by adipose tissues can regulate the levels of NPY and POMC.^169^, ^170^, ^171^, ^172^ The hypothalamus–serotonin axis plays an important role in cachexia–anorexia syndrome.^173^

Growth differentiation factor 15 (GDF15) is a member of the TGF‐β family and produces anorexia by directly acting on the feeding center of the brainstem.^174^ In patients with cancer cachexia, the level of circulating GDF15 increases in the early stage of cachexia and continues to rise with the progression of cachexia.^175^ In addition, experiments have shown that circulating GDF15 levels in tumor patients with cachexia are significantly higher than those in tumor patients without cachexia.^176^ GDF15 has a strong effect on anorexia and can affect metabolism, potentially causing skeletal muscle wasting and lipolysis in cancer cachexia.^177^ Animal experiments showed that mice injected with recombinant GDF15 or transplanted with GDF15 overexpression tumor showed decreased food intake and weight loss, whereas mice with cachexia treated with an anti‐GDF15 antibody show weight gain and muscle and adipose tissue recovery.^178^ This effect is mediated by a glial cell‐derived neurotrophic factor family receptor alpha‐like (GFRAL),^179^ which is mainly expressed in nerve cells in the area postrema and nucleus of the solitary tract and can regulate energy balance during inflammation, aging, and nutritional imbalance.^180^, ^181^ Although the mechanism of GDF15‐induced muscle wasting and lipolysis of cachexia is unclear, circulating GDF15 levels may be the biomarkers of cancer cachexia. In short, the GDF15–GFRAL axis controls body weight through central pathways (food intake) and peripheral pathways (lipid oxidation and muscular wasting), especially in cancer cachexia.^182^ The GDF15–GFRAL axis is a promising target for the study of cancer cachexia.

Neuroendocrine disorders play an important role in the occurrence and development of cancer cachexia. This endocrine disorder is the result of the interaction between the hypothalamus and multiple organs. During cancer cachexia, cytokines such as TNF‐α and IL‐6 enter the central nervous system and promote the wasting of surrounding tissues, such as muscles through the hypothalamus–pituitary–adrenal and hypothalamus–pituitary–gonad axes.^183^, ^184^, ^185^

### 4.2 Peripheral nerve and cancer cachexia

Increases in sympathetic tension and continuous increase in basal metabolic rate in patients and mouse models with cancer cachexia have been described as the key energy consumption mechanism during cancer cachexia.^183^ Since the increased thermogenesis of brown adipose tissue (BAT) in cachexia mice was reported for the first time, it has been reported in a variety of cachexia mouse models.^186^ In fact, recent studies have shown that the conversion of WAT to BAT increases the energy consumption of tumor‐associated cachexia. Sympathetic nerves (SNS) facilitate the conversion of WAT to BAT, SNS acts through sympathetic ganglia throughout the body to regulate end organs by releasing norepinephrine at synapses and adrenaline from the adrenal medulla into the circulation. These neurotransmitters participate in lipolysis of WAT by increasing heart rate and cardiac contractility, and induce nonexercise thermogenesis in BAT by decoupling electron transport in mitochondria from ATP production, thereby increasing the metabolic rate.^141^ Although sympathetic activation is significantly involved in the progression of cancer cachexia, the exact mechanism of sympathetic involvement in cachexia is not fully understood.^187^ A recent report by Kim et al.^188^ showed that the brain–fat axis precisely and rapidly activates lipolysis during bacterial infection, and this effect is entirely dependent on the activation of TNF receptors in the hypothalamic arcuate nucleus and increased sympathetic action. Given that TNF, also known as cachexia factor, is considered an important mediator of central nervous system inflammation during cancer cachexia, this brain–fat axis may be involved in adipose tissue remodeling and emaciation observed during cancer cachexia. Chronic inflammation caused by cachexia may induce the continuous activation of the brain–fat axis through hypothalamic TNF signals, leading to the progressive lipolysis of cachexia.^183^ Patients with cancer cachexia show significant decrease in heart rate variability, which is a result of sympathetic activation.^189^, ^190^, ^191^ The brain–fat heart axis plays an important role in maintaining heart rate variability and GDF15 is the key regulator of this axis.^176^, ^177^, ^192^ The expression of GDF15 is upregulated in many rodent cachexia models and patients with cancer. Increased sympathetic activity during cancer cachexia can increase the outputs of glucose and adipose tissues from the liver, increasing energy consumption and resulting in weight loss.^193^ Some studies confirmed that epinephrine can increase mitochondrial respiration,^194^ and blocking β3‐adrenoceptor can alleviate lipolysis, the browning of WAT, and cachexia‐related weight loss.^78^

### 4.3 Innervation of tumor and cancer cachexia

Cells secrete exosomes, which carry nucleic acids, lipids, proteins, and metabolites, and contribute to communication intercellular communication.^195^ Exosomes released by tumors mediate the innervation of tumors, which can regulate local immune response and plays an important role in the progression of cancer. For example, in the context of cancer cachexia, WAT actively secretes exosomes containing microRNA, which may regulate the inflammatory process in tissues and immune cells.^196^ Exosomes from adipose stromal cells reduce proliferation rates of stimulated T lymphocytes in vitro. In the case of CD8+ T cells, exosomes significantly reduce percentages of terminally differentiated effector memory cells. Concerning CD4+ T cells, exosomereduce percentages of effector‐memory cells and significantly increase percentages of central memory cells.^197^ In addition to altering the immune system, exosomes can lead to muscle wasting in the context of cancer cachexia.^198^, ^199^, ^200^ Recent studies have confirmed HSP70 and HSP90, GDF15 and Prolyl 4‐hydroxylase to be responsible for muscle wasting of cancer cachexia in mice.^201^, ^202^, ^203^ Using conditioned medium from cachexia‐induced tumor cell lines, including LLC and colon C26, it was found that exosomes containing heat shock proteins 70 (HSP70) and heat shock protein 90 (HSP90) can cause muscle wasting. The release of heat shock proteins HSP70 and HSP90 directly induced muscle catabolism in LLC cachexia model. Mechanically, this is achieved by activating TLR4 and p38‐MAPK pathway in muscle cells, which can be inhibited by neutralizing or silencing HSP70 and HSP90 in LLC cells.^201^ A similar effect was seen in a pancreatic cancer xenograft model. Zinc transporter ZIP4 stimulates exosomes release via RAB27B GTPase, and mice carrying both ZIP4 knockout pancreatic cancer cell xenografts lost less weight and survived longer compared with control mice.^204^ In an LLC tumor model carrying microRNA‐21, tumor‐derived exosomes stimulate apoptosis of myoblasts via TLR7 signaling and lead to muscle wasting.^205^ Although tumor innervation may be closely related to cancer cachexia, the mechanism by which tumor nerve‐derived factors trigger muscle wasting and lipolysis in cachexia remains unclear, which need more research. The interaction between neural and immune is evolutionarily conserved, it is very important for dynamic balance. Tumors may promote their own innervation to inhibit immune response and promote tumor tolerance, disease progression, and spread.^206^ A neuroimmune circuit exists in interorgan communication.^207^ The crosstalk of multiple organs in neuroimmunity during cancer cachexia accelerates the process of cachexia.

In short, the nervous system adjusts homeostasis in the body in the early stage of the tumor to resist tumor progression, but this function is disrupted by the continuous attack of inflammatory factors released by hosts and tumor cells during tumor progression and the occurrence of cancer cachexia. Owing to the abnormal function of the hypothalamus and increase in sympathetic tension, anorexia occurs and energy consumption increases, and thus the process of cancer cachexia is accelerated.

## 5 MULTIPLE ORGANS IMMUNE IMBALANCE IN CANCER CACHEXIA

The suppression of the immune system is a characteristic of cancer cachexia. The imbalance of the multiple organs immune system is the result of the development of tumors. Immune cells circulate in the body and transfer to places where they need to perceive and respond to stimuli, to restore the dynamic balance of the tissue.^207^ The immune system attacks tumor cells and inhibits tumor growth, but becomes ineffective in killing tumor cells as tumor progresses, and its balance is disrupted. Excessive immune activation aggravates damage and accelerates the process of cancer cachexia. Many immune cells play a role in cancer cachexia, especially in promoting muscle wasting in cancer cachexia.^208^ For example, tumor‐induced IL‐6 impairs the ketogenic response to reduced caloric intake, resulting in a systemic metabolic stress response that blocks anticancer immunotherapy.^209^ But there are some immune cells that can prevent cancer cachexia progression. For example, IL‐4 has been shown to reduce skeletal muscle wasting in a mouse model of C26 cachexia.^210^ A study show an immune cell subset that promotes protection from cachexia, for example, CD4+ can relieve the symptoms of cachexia and the decrease of CD4+ will aggravate the symptoms of cancer cachexia.^211^ Targeting immune cells may play a role in the treatment of cancer cachexia, but further research is needed. Disorders of immune cells lead to imbalances in immune organs and the imbalance of immune organs in cancer cachexia is mainly reflected in the bone marrow and spleen (Figure 3).

### 5.1 Bone and cancer cachexia

In human and animal models, loss of muscles or bones is related to aging, disuse, and tumors, and the coordination of muscle and bone mass is realized by mechanical signals generated by muscle force.^212^ Bones and Scheckel muscles interact with one another. Muscle‐derived factors IGF‐1 and fibroblast growth factor 2 can stimulate bone formation,^213^ and bone‐derived factor Indian hedgehog can promote muscle growth.^214^ On the basis of the deepening understanding of the physiological and molecular mechanisms in muscles and bones, muscle and bone mass may be lost simultaneously during cachexia because many signal pathways that induce muscle wasting promote bone loss.^215^ Growing evidence shows that mediators associated with the pathogenesis of skeletal muscle and fat loss in cancer cachexia induce bone mass loss in a similar way.^216^ In addition, some bone‐derived factors affect muscle wasting. Some studies showed that the release of osteoclast‐mediated potential TGF‐β from the bone matrix can affect the intracellular calcium signal and the skeletal muscle function of bone metastatic mice,^217^ providing a novel therapeutic target for the treatment of cachexia. The study of bone–muscle crosstalk during cancer cachexia may provide a novel method for the treatment of cancer cachexia, but the exact mechanism needs to be further studied.

The relationship between the bone marrow and cancer cachexia is reflected in immunity. In cancer cachexia, tumor tissues or host cells interact with other tissues or organs by secreting cytokines. Immune cells differentiated from bone marrow hematopoietic stem cells play an important role in systemic inflammation and may be related to the interaction between organs during cancer cachexia. Bone loss occurs in cancer cachexia animal models, accompanied by decrease in bone marrow mesenchymal stem cells, and the number of bone marrow mononuclear cells begins to decrease significantly before significant loss of muscles and fat.^218^ Myelogenous suppressor cells (MDSCs) are induced by proinflammatory cytokines and represent immature myeloid cell populations at different stages. They exist not only in the bone marrow but also in secondary lymphoid organs, such as the spleen and lymph nodes. The number of MDSCs in secondary lymphoid organs increases with inflammatory stimulation.^219^ Increase in the number of MDSCs in tumor‐carrying hosts renders susceptible to cachexia by inhibiting acquired immunity,^220^, ^221^, ^222^ but the specific mechanism needs to be further studied.

### 5.2 Spleen and cancer cachexia

Spleen as an immune organ that plays an important role in cancer cachexia, but studies on the spleen in cancer cachexia are few. Some studies confirmed that cancer cachexia can be mediated by IL‐6‐like cytokines in the spleen,^223^ which can promote lipolysis. In LLC cachexia models, the authors found that the spleen expresses some cytokines related to cachexia, such as gp130. Splenic enlargement has been reported in cachexia models.^224^ Splenomegaly is generally a manifestation of hyper function, including extra medullary hematopoiesis and immune hyperplasia. The high catabolism and energy consumption of enlarged spleen aggravate cachexia process during cancer cachexia.^225^

The immune system plays an important role in killing tumor cells at the initial stage of tumors, but tumors can evade immune reactions, and the continuously released immune factors enter the circulatory system, some of which play a role in promoting tumor development. The immune disorder of the bone marrow and spleen aggravates the process of cancer cachexia.

## 6 TUMOR ENVIRONMENT AND CANCER CACHEXIA

### 6.1 TME promotes the development of cancer cachexia

TME consists of cancer cells, various stromal cell populations such as infiltrating immune cells, neutrophils, fibroblasts, and adipocytes, as well as extracellular matrix (ECM) components, soluble factors, and signaling molecules produced by these cells. Growing evidence suggests that TME plays an important role in cancer progression and tumor‐induced cachexia by producing a variety of cachexia factors, such as macrophages, neutrophils, and fibroblasts that can produce IL‐6, TNF‐α, and other cachexia factors aggravate cachexia TME.^226^ These circulating factors can act directly with muscle cells by activating precachexia programs in muscle, promoting muscle catabolism or inhibiting protein synthesis pathways, or they can lead to muscle wasting through metabolic reprogramming of secondary organs. TME is involved in immune cell activation and recruitment, angiogenesis, and ECM remodeling, all of which can lead to tumor progression.^227^ In addition, TME is a site of local inflammation, leading to increased systemic inflammation and oxidative stress in patients with cancer cachexia.^228^ The secreted products of TME cells can affect other organs and systems. The communication and interaction among tumors, TME and multiple organs constitute the macroenvironment of cancer cachexia and promote development of cancer cachexia.

### 6.2 Cancer cachexia aggravates the disturbance of TME

There is a possibility that tumors are getting benefited by inducing cachexia. A rapidly growing tumor utilizes glucose, fatty acid, lactate, and amino acids, to manage excess energy requirement and amino acids to achieve rates of proliferation.^82^ To a certain extent, this may indicate that cancer cachexia can promote the development of tumors. Cancer cells can functionally sculpt their microenvironment through the secretion of various cytokines, chemokines, and other factors.^229^ For example, neutrophils may act as tumor‐promoting leucocytes by producing TGF‐β and IL‐10, thereby inducing regulatory T‐cell pathways and matrix metalloproteinases in the TME.^230^ In the context of cancer cachexia, various factors released by the tumor and the host accelerate the disturbance of TME.

## 7 TREATMENT OF CANCER CACHEXIA

**FIGURE 4:**
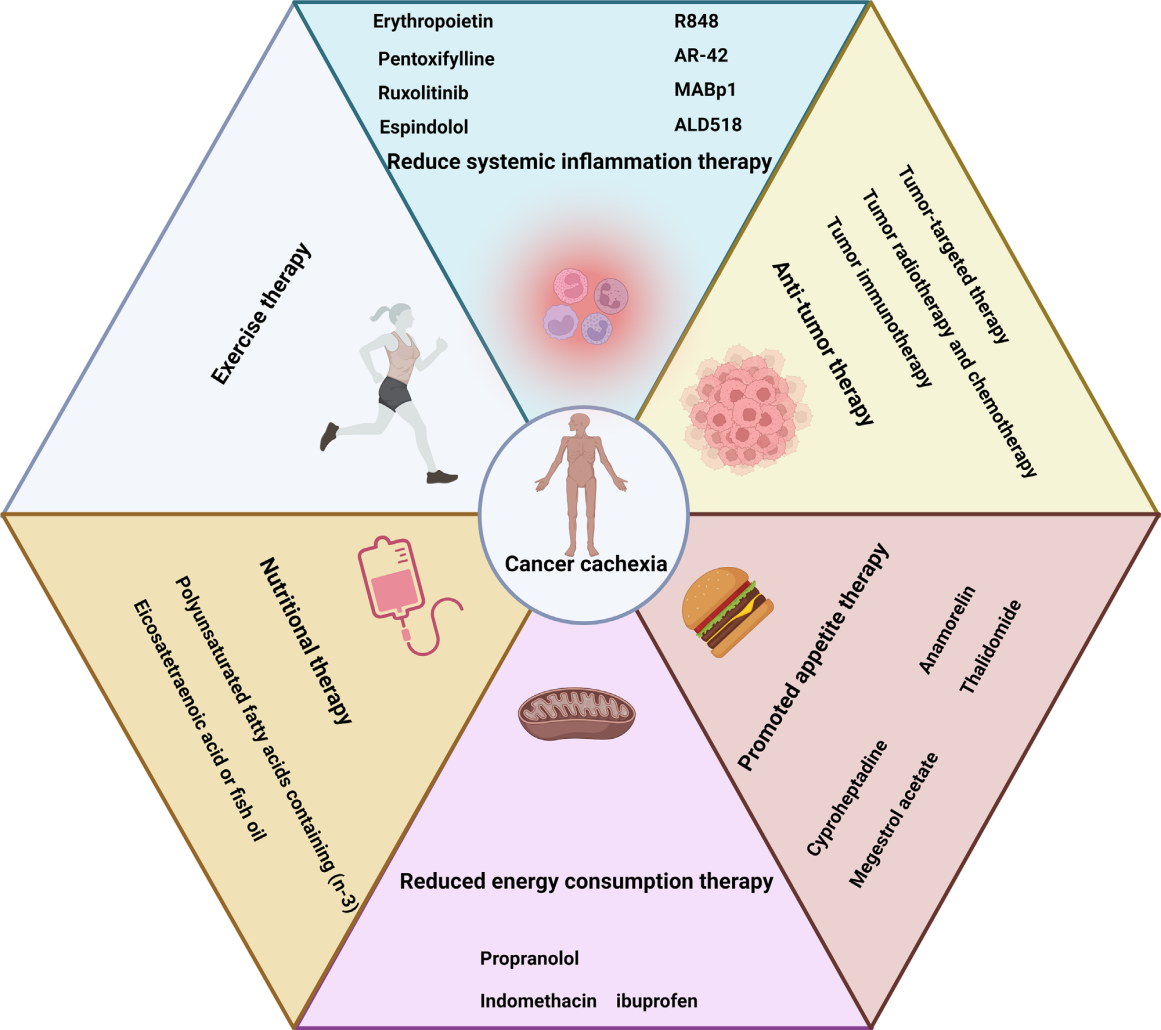
Possible treatment strategies for cancer cachexia. Created with BioRender.com.

Cachexia is the most serious complication of tumors and an important cause of disability and death. It is associated with significant decrease in the survival rate of a patient with cancer.^231^ No approved treatment method is currently available.^232^ The treatment of cancer cachexia requires the participation of multiple disciplines and modes.^37^, ^233^, ^234^, ^235^, ^236^ Except antitumor therapy, methods for the treatment of cachexia include the promotion of anabolic metabolism and anticatabolism, use of appetite stimulants, and nutritional interventions, drug therapies that stimulate appetite and reduce inflammation, nutritional therapy that increases energy and protein intake, and exercise therapy are available. The cooperative treatment of cancer cachexia is related to the fact that cancer cachexia is a multiple organs syndrome. The treatment of cachexia improves multiple organs function and reduce metabolic, neurological, and immune disorders and can thus improve the survival rate and quality of life of patients with cancer cachexia. Table 2 and Figure 4 provide the classification of treatments for antitumor and improvement of cancer cachexia in recent years.

**TABLE 2:** Treatment strategies of supportive therapy for cancer cachexia

	Drugs/class	Effects	Possible mode of action	Reference
Reduce systemic inflammation therapy	Espindolol (nonspecific β 1/β2 adrenergic receptor antagonist)	pro‐anabolic, anti‐catabolic, and appetite‐stimulating actions	central 5‐HT 1A and partial β2 receptor agonist effects	^237^
	Ruxolitinib (JAK1/ JAK2 inhibitor)	Combined with capecitabine to improve overall survival rate	Inhibit tumor angiogenesis, control disease progression and improve the survival rate	^238^
	Pentoxifylline (methylxanthine derivative)	Inhibit systemic inflammatory response and improve quality of life	Inhibition of phosphodiesterase	^239^
	Erythropoietin	Inhibit weight loss	Reduced the production of IL‐6	^240^
	ALD518 (Humanized monoclonal antibodies against Human IL‐6)	Improves grip strength and fatigue	against IL‐6	^241^
	MABp1 (Humanized monoclonal antibodies against IL‐1a)	Prolonged the median overall survival time	against IL‐1a	^242^
	AR‐42 (Histone deacetylase inhibitor)	Protects loss of muscle and adipose tissue	Inhibit the production of inflammatory cytokines and proteins related to cancer cachexia.	^243^
	R848 (TLR7/8 agonist)	Reshape the tumor immune microenvironment and improve the survival rate.	Unclear, may have something to do with improving immunity.	^244^
Reduce energy consumption therapy	Propranolol (selective β‐2 receptor blocker)	reverses the hyper metabolism	block epinephrine circulation	^245^
	Indomethacin, ibuprofen. (cyclooxygenase inhibitors)	Reduce resting energy consumption	against cachexia‐related inflammatory factors	^246^
Nutritional therapy	Polyunsaturated fatty acids containing (n‐3)	Resist muscule wasting and improve the survival rate	Unclear	^247^
	Eicosatetraenoic acid or fish oil	Reduce muscle wasting.	Uclear, may be related to against cachexia‐related inflammatory factors.	^248^
	Leucine	Reduce muscle wasting	Improve mitochondrial function	^249^
Promote appetite therapy	Anamorelin (Ghrelin simulator)	Increase appetite and weights	Increase the secretion of growth hormone	^250^
	Megestrol acetate (progesterone analogue)	increase the appetite and body weights	Increase the release of neuropeptide Y in hypothalamus, decrease and inhibit proinflammatory cytokines	^251^
	Thalidomide	Improve appetite and resist weight loss	Reduce TNF‐α	^252^
	Cyproheptadine (a serotonin inhibitor)	produces mild appetite stimulation	Unclear, may be related to the inhibition of hypothalamic satiety center	^253^

### 7.1 Antitumor therapy

Crosstalk between tumor cells and organs promotes the occurrence of cachexia.^254^ Treating and delaying the progression of tumor can prolong the process of cachexia and delays cancer cachexia treatment. Tumor treatments include targeted therapy, radiotherapy, chemotherapy, and immunotherapy.

#### 7.1.1 Tumor‐targeted therapy

The original intention of tumor‐targeted research is to treat tumors and reduce adverse reactions. Owing to scientific advances, tumor‐targeted therapies that do not aggravate cachexia have increased, such as tumor hyperthermia and magnetic therapies.

#### 7.1.2 Tumor radiotherapy and chemotherapy

Radiotherapy and chemotherapy can reduce tumor volume and improve the survival rates of patients but can induce muscle wasting and host fatigue,^255^ especially chemotherapy, which triggers cachexia by relieving the synergistic effects of histone modifying enzymes.^256^ Nevertheless, *Redd1* gene deletion has a preventive effect on chemotherapy‐induced muscle wasting and weakness in mice.^257^ Supplementation with exogenous IGF‐1 can reduce cisplatin‐induced muscle atrophy in mice.^258^ In the future, solving the contradiction among radiotherapy, chemotherapy, and side effects is the key link in the treatment of cachexia.

#### 7.1.3 Tumor immunotherapy

Tumor immunotherapy has been a focus of research in recent years and has greatly changed the tumor treatment. Targeting the immune system for the identification and destruction of cancer cells provides many patients with the possibility of depth, long‐term remission, and potential cure.^259^ Not all tumors are suitable for immunotherapy. Finding novel ways to expand the accessibility of immunotherapy to more tumor types is still a challenge that tumor immunotherapy needs to overcome.

### 7.2 Supportive therapy

Alleviating cancer cachexia, correcting metabolic, neural, immune disorders of organs, and maintaining its normal function can increase a host's tolerance to radiotherapy, chemotherapy, and antitumor drugs. Specific measures include reducing systemic inflammatory response in cancer cachexia, promoting appetite, increasing nutritional intake, and moderate exercise.^25^, ^260^

#### 7.2.1 Reduce systemic inflammation therapy

A typical feature of cancer cachexia is increase in systemic inflammatory response, which leads to tumor growth and organ failure through a cascade of inflammatory factors.^261^ The treatment of cachexia‐related inflammatory factors has been a focus of research.^262^ As a powerful HT‐1A receptor agonist in an animal study, espindolol can bind to HT‐1A receptors in the brain and increase muscle content and body weight.^237^ Maintaining the expression of Hand2 in the central nervous system of older mice can prevent inflammation, which has potential use in cachexia treatment.^263^ As a JAK1/JAK 2 inhibitor, ruxolitinib combined with capecitabine can improve the overall survival rates of patients with metastatic pancreatic cancer and systemic inflammation.^238^ As a methyl xanthine derivative, pentoxifylline inhibits systemic inflammation and TNF‐α by inhibiting phosphodiesterase. The efficacy of pentoxifylline in cancer cachexia has not been confirmed.^239^ Erythropoietin inhibits the production of IL‐6 in preclinical cancer cachexia model.^240^ IL‐6 is a potent mediator of cancer cachexia muscle wasting. Humanized anti‐IL‐6 antibodies and drugs that inhibit GDF8^264^ play an important role in the treatment of cancer cachexia. In a clinical trial, humanized monoclonal antibody ALD518 against human IL‐6 significantly improved grip strength and alleviated fatigue and cancer‐associated cachexia in patients with advanced non‐small‐cell lung cancer.^241^ MABp1, another humanized monoclonal antibody against cytokine IL‐1a, prolongs median overall survival in cachexia patients with advanced colorectal cancer.^242^ AR‐42, a newly discovered histone deacetylase inhibitor, inhibits the production of various inflammatory cytokines and proteins related to cancer cachexia and finally shows the therapeutic potential for muscle and fat loss in mice with cancer cachexia.^243^ TLR7/8 agonist R848 improves cachexia in the cachexia model of pancreatic ductal adenocarcinoma (PDAC) mice.^244^

#### 7.2.2 Reduced energy consumption therapy

Host resting energy increases in cancer cachexia, and this effect is related to muscle wasting. Reducing resting energy consumption is an important part of cancer cachexia treatment. By inhibiting sympathetic activation during cancer cachexia, drugs such as propranolol, can selectively block β‐2 receptors, block epinephrine circulation, and reverses the hyper metabolism in about half of patients with cancer.^245^ Cyclooxygenase inhibitors (indomethacin and ibuprofen) reduce resting energy consumption in patients with solid tumors.^246^

#### 7.2.3 Nutritional therapy

Although weight loss due to cancer cachexia cannot be completely reversed by nutritional supplementation^265^ and some people show concern about possible nutritional supplementation‐induced tumor growth,^266^ some studies found that patients with cancer cachexia who receive active refeeding may have refeeding syndrome in the first 2–3 weeks.^267^ Nevertheless, the nutritional treatment of cancer cachexia still occupies an essential position.^268^, ^269^, ^270^, ^271^ An updated meta‐analysis in 2018 indicated that dietary counseling and oral nutritional supplements are associated with weight improvement in patients receiving chemotherapy or radiotherapy.^272^ In a CT26 mouse model that underwent chemotherapy, specific nutritional support therapy reduced systemic inflammation and maintained muscle function and physical activity levels without promoting tumor growth or affecting the efficacy of chemotherapy.^273^ A prospective experimental study confirmed that enteral nutrition support with polypeptide formula contributes to the body weight stability of patients with advanced PDAC cachexia.^274^ Moreover, oral dietary supplements of polyunsaturated fatty acids containing (n‐3) can resist muscle atrophy and improve the survival rates of patients with advanced lung cancer.^247^ Anti‐inflammatory supplements, such as eicosapentaenoic acid or fish oil reduce muscle wasting,^248^ and dietary leucine supplementation can reduce cachexia muscle wasting by improving mitochondrial function.^249^ Cannabinoids interact with endorphin receptors, disrupt IL‐1 synthesis, activate cannabinoid receptors in leptin neural circuits, and inhibit prostaglandin synthesis and can thus increase energy intake and improve nitrogen balance in the body.^275^ However, it is not recommended for the treatment of cancer cachexia because it has severe adverse effects on the central nervous system, such as hallucinations, dizziness, and psychosis.

#### 7.2.4 Promoted appetite therapy

Anorexia is a typical clinical manifestation of cancer cachexia. Promoting appetite is the way that enables patients to absorb necessary nutrients. Ghrelin, which is mainly secreted by the gastrointestinal tract, is a promising option for the treatment of cancer cachexia. It can promote appetite and anabolism.^276^ Anamorelin hydrochloride, one of the drugs, can significantly increase the appetite and body weight of patients with cancer cachexia.^250^ The progesterone analog megestrol acetate reduces the levels of proinflammatory cytokines by increasing the release of NPY in the hypothalamus while improving the appetite and weights of patients with cachexia.^251^ Thalidomide can alleviate the symptoms of appetite loss and resist weight loss due to cancer cachexia.^252^ Cyproheptadine is a serotonin inhibitor that produces mild appetite stimulation and a certain degree of sedation.^253^

#### 7.2.5 Exercise therapy

Exercise therapy plays an important role in the treatment of cancer cachexia, thereby attracting considerable interest in recent years.^277^, ^278^, ^279^, ^280^, ^281^, ^282^, ^283^ Exercise can enhance metabolite interactions among the heart, liver, and muscle,^284^, ^285^ indicating that exercise can regulate metabolic homeostasis among multiple organs. In terms of the benefits of exercise on the heart, exercise can reduce myocardial necrosis, fibrosis, and inflammation, reduce the levels of TGF‐β1 and BNIP3 mRNA, and increase the level of mitochondrial complex IV protein, resulting the effect of agonist ventricular remodeling.^126^ In terms of the effects of exercise on the liver and muscles, exercise can reduce the symptoms of cancer cachexia by regulating muscle metabolism, insulin sensitivity, and inflammation.^286^ Exercise can increase the activities of antioxidant enzymes and their antioxidative effects, preventing muscle catabolism caused by reactive oxygen.^287^ By reducing protein decomposition, exercise can enhance insulin sensitivity and promote protein synthesis during cancer cachexia. In addition, it can minimize tumor growth after chemotherapy and can reduce muscle loss caused by chemotherapy.^288^ Resistance exercise (weight training) can increase the muscle mass of bedridden patients^262^ and may have a therapeutic effect on patients with limited activity in the late stage of cancer cachexia. In short, moderate exercise can reduce muscle wasting in patients with cancer cachexia by reducing protein decomposition and oxidative stress caused by systemic inflammation. Antineoplastic drugs, radiotherapy, and chemotherapy are double‐edged swords, and tumor treatment causes damage to patients. Exercise therapy is a nondrug treatment of cancer cachexia and is expected to be fully used in clinics in the future.

The above strategies are far from exhaustive and Table 3 lists the completed clinical trials related cancer cachexia in recent years. Owing to increasing attention devoted to cancer cachexia and the high demand for the quality of life in the advanced tumor stage, drugs for the symptomatic treatment of cancer cachexia have increased. The rapid development of antitumor therapy is likely to offer breakthroughs in the treatment of cancer cachexia.

**TABLE 3:** Currently completed clinical studies on cancer cachexia

Study type	Start time(year)	Intervention	Study group and finding	Source (reference)
Clinical Trial	January 2000	Insulin, isophane	Insulin treatment significantly stimulated carbohydrate intake, decreased serum‐free fatty acids, increased whole body fat, particularly in trunk and leg compartments, whereas fat‐free lean tissue mass was unaffected. Insulin treatment improved metabolic efficiency during exercise, but did not increase maximum exercise capacity and spontaneous physical activity.	NCT00329615^144^
Clinical Trial Phase II	January 2000	Megestrol; Exercise	Unknown	NCT00004912
Clinical Trial Phase III	March 2000	Megestrol acetate eicosapentaenoic acid (EPA)	This EPA supplement, either alone or in combination with MA, does not improve weight or appetite better than MA alone.	NCT00031707^289^
Clinical Trial Phase III	September 1, 2000	Megestrol	Unknown	NCT00031785
Clinical Trial Phase II	April 2003	Infliximab; gemcitabine	Adding infliximab to gemcitabine to treat cachexia in advanced pancreatic cancer patients was not associated with statistically significant differences in safety or efficacy when compared with placebo.	NCT00060502^290^
Clinical Trial Phase III	May 2003	Etanercept	Etanercept, as prescribed in the current trial, does not appear to palliate the cancer anorexia/weight loss syndrome in patients with advanced disease.	NCT00046904^291^
Clinical Trial Phase II	June 2003	Cyproheptadine	Cyproheptadine hydrochloride is a safe and effective way to promote weight gain in children with cancer/treatment‐related cachexia	NCT00066248^292^
Clinical Trial Phase II	December 2003	N‐acetylcysteine	N‐acetylcysteine strongly enhanced the increase in knee extensor strength and significantly increased the sum of all strength parameters if adjusted for baseline arginine level as a confounding parameter.	NCT00196885^293^
Clinical Trial Phase II	June 2004	A Fish Oil	Unknown	NCT00094562
Clinical Trial Phase III	December 2004	Creatine	Unknown	NCT00081250
Clinical Trial I	February 2006	A low antioxidant diet	Unknown	NCT00486304
Clinical Trial Phase II	September 2006	RC‐1291	Unknown	NCT00378131
Clinical Trial	July 2007	Eicosapentaenoic Acid	Change in Serum Albumin Number of Participants With Proteasome Activity That Was Inhibited in the Range of 6%‐29%.	NCT00815685
Clinical Trial	March 2009	Lenalidomide	Unknown	NCT01127386
Clinical Trial Phase II	March 2009	APD209	Unknown	NCT00895726
Clinical Trial Phase I/II	June 2009	Ghrelin	Ghrelin is well tolerated and safe in patients with advanced cancer. For safety, tolerance, and patients' preference for treatment, no difference was observed between the lower‐ and upper‐dose group.	NCT00933361^294^
Clinical Trial	September 2010	Exercise training	Unknown	NCT01136083
Clinical Trial Phase II	April 2011	MT‐102	Unknown	NCT01238107
Clinical Trial Phase III	July 2011	Anamorelin HCl	Over the entire 0–24w treatment period, body weight and symptom burden were improved with anamorelin.	NCT01395914^295^
Clinical Trial Phase II	August 2011	BYM338	Unknown	NCT01433263
Clinical Trial Phase II/III	April 2012	Omega‐3	Echium oil effectively increased erythrocyte EPA and GLA FAs in H&N cancer patients. It failed however to protect against weight loss, or improve nutritional parameters	NCT01596933^296^
Clinical Trial	May 2012	Activin A	Increased circulating concentrations of ActA may contribute to the development of cachexia in cancer patients.	NCT01604642^297^
Clinical Trial	October 2014	Cachexia Acupuncture‐A	Unknown	NCT02148159
Clinical Trial Phase IV	April 2015	n‐3 LCPUFAs	Unknown	NCT04699760
Clinical Trial	June 29, 2016	12 Week Home‐based Exercise Intervention	Unknown	NCT04802486
Clinical Trial	November 2016	Cannabis Capsules	Despite various limitations, this preliminary study demonstrated a weight increase of ≥10% in three out of 17 (17.6%) patients with doses of 5 mg×1 or 5 mg×2 capsules daily, without significant side effects. The results justify a larger study with dosage‐controlled cannabis capsules in CACS.	NCT02359123^298^
Clinical Trial I	October 16, 2017	Onivyde; 5‐FU	Unknown	NCT03207724
Clinical Trial	May 23, 2018	Vitamin D	Unknown	NCT03144128
Clinical Trial Phase II/III	March 26 2018	Mirtazapine	Unknown	NCT03254173
Clinical Trial Phase III	March 26, 2019	Mirtazapine; Megestrol	Unknown	NCT03283488
Clinical Trial Phase II	February 13, 2020	Curcumin	The curcumin add‐on resulted in a significant increase in muscle mass than standard nutritional support. Furthermore, it may improve and delay a decrease in the other body composition parameters, handgrip strength, and absolute lymphocyte count. Curcumin was safe and well tolerated. This constitutes an unmet need for clinical trials.	NCT04208334^299^

Date sources: https://www.clinicaltrials.gov/.

## 8 SUMMARY AND PROSPECT OF THE FUTURE

Cancer cachexia has attracted widespread interest because of its high morbidity and mortality. Improvements in tumor treatment have increased the survival times of patients with cancer. The evolution of the relationship between tumors and organs leads to the process of cancer cachexia. Metabolic, neural, and immune functions of organs have complicated relationships with the occurrence and development of cancer cachexia. Focusing on cancer cachexia and changes in organs is the key to the improvement of the survival rates and quality of life of patients with advanced cancer. More in‐depth research is needed in the future.

At present, we do not have a deep understanding of the mechanism of cancer cachexia, especially the relationship between cancer cachexia and the imbalance of multiple organs homeostasis. Moreover, a standardized method for the diagnosis and treatment of cachexia is lacking. Nevertheless, developments in organoids, single‐cell technology, and metabolomics^300^ will help us in further exploring the relationship among tumors, the TME, cancer cachexia, and multiple organs homeostasis imbalance and to determining core signals and molecular mechanisms. The integrated application of different methods for the treatment of cancer and cancer cachexia, including diet and exercise, has deepened our understanding of the relationship between cancer cachexia and multiple organs homeostasis imbalance. The systematic analysis and understanding of the relationship between cancer cachexia and multiple organs imbalance will prompt us to rerecognize cancer cachexia and discover novel strategies for cancer cachexia treatment and for increasing the survival rates and quality of life of patients with cancer cachexia from the perspective of expanded TME.

## FUNDING

This research did not receive any specific grant from funding agencies in the public, commercial, or nonprofit sectors.

## AUTHOR CONTRIBUTIONS

Yong‐Fei Wang and Zi‐Yi An contributed to the conception of the study and wrote the initial draft. Dong‐Hai Lin reviewed the manuscript and provided suggestions for revision. Wei‐Lin Jin helped perform the analysis with constructive discussions. All authors read and approved the final manuscript.

## CONFLICT OF INTEREST

The authors declare that they have no competing interests.

## ETHICS STATEMENT

None.

## Data Availability

None.
